# O21 A case of thromboembolic ischaemic cardiomyopathy and intracardiac thrombus in a patient with antiphospholipid syndrome

**DOI:** 10.1093/rap/rkab067.020

**Published:** 2021-10-19

**Authors:** Edward Alveyn, Arti Mahto

**Affiliations:** Kings College Hospital, London, United Kingdom

## Abstract

**Case report - Introduction:**

Commonly found in association with lupus, antiphospholipid syndrome (APLS) is a potentially life-threatening disease of which an understanding is essential for rheumatologists. In addition to well-recognised sequelae such as pulmonary embolism and obstetric complications, APLS can provoke thrombi ranging from microscopic to massive in size in a wide range of arterial and venous territories. We present the case of a young woman with APLS who suffered significant morbidity as a result of intracardiac and coronary thromboembolism shortly after becoming pregnant and switching anticoagulant therapy, highlighting the importance of vigilance and investigation for rarer thromboses in APLS patients.

**Case report - Case description:**

A 34-year-old woman with known APLS and 5 weeks pregnant was admitted to hospital with a history of headache, nausea/vomiting and mild photophobia followed by fever, shortness of breath, confusion, pleuritic chest pain and lower limb swelling. She had commenced enoxaparin in place of warfarin on becoming pregnant. Examination was suggestive of cardiac failure. Troponin and NTproBNP were markedly elevated, without ischaemic ECG changes. A brain CT venogram was reported as normal, but echocardiogram revealed a dilated LV with reduced ejection fraction (39%), inferior and lateral wall hypokinesia and possible LV thrombus. She was treated initially for myocarditis (presumed viral or autoimmune) and received antibiotics given her raised WCC and CRP. Treatment dose enoxaparin was continued.

Bloods revealed anaemia, thrombocytopenia, and positive immunology: cardiolipin IgG 123U/ml, IgM 612, anti-B2GP1 IgG 19/IgM 607, ANA (1/320) and RNP 70. C3 was normal (0.8) and C4 low (0.03). A livedoid rash consistent with APLS was present on the trunk, but there were no other clinical manifestations of connective tissue disease.

Repeat CT venogram performed after the patient reported worsening headaches revealed a small tentorial subdural haematoma, resulting in the reversal of enoxaparin with protamine. Later review of these images suggested a stable 5mm haematoma that was present on the earlier scan, and enoxaparin was recommenced.

Cardiac MRI revealed extensive infarct with contained LV wall rupture. Coronary angiography showed normal vessels. LVEF on repeat echocardiogram fell to 28%. Surgical pregnancy termination was performed in accordance with patient wishes, with subsequent reversion to warfarin anticoagulation.

Repeat MRI showed thinned anterior/lateral LV walls, evidence of transmural myocardial fibrosis and residual laminar thrombus, and bubble echo demonstrated no PFO. The patient was ultimately managed for presumed microembolic myocardial infarction with resulting heart failure, and has been referred to a cardiac transplant centre.

**Case report - Discussion:**

This case highlights the potential risk associated with a relatively common scenario: anticoagulant switching in females with APLS at the start (or in anticipation) of pregnancy. In this case our patient started enoxaparin 80mg BD 48 hours after discontinuing warfarin, developing symptoms consistent with intracerebral thrombosis shortly afterwards, followed by those of heart failure.

The possible diagnoses on the basis of the patient’s initial presentation were numerous, and she was appropriately investigated in the first instance for a possible cerebral thrombotic event with cranial CT and venogram. On development of cardiorespiratory symptoms, there was a delay in requesting investigations (troponin, BNP) that may have pointed towards myocardial pathology, and once these investigations were noted to be abnormal the patient was managed as a probable myocarditis in keeping with most other patients of her age without a significant past medical history.

Perhaps insufficient diagnostic weight was given to her known thrombophilia and recent medication change, which may have prompted closer review of her brain imaging leading to earlier detection of the subdural haematoma. It may also have led to more rapid investigation for possible thrombus elsewhere via earlier echo, CTPA or cardiac MRI. The latter investigation was ultimately crucial in definitively showing myocardial injury to be the result of infarction rather than inflammation, where prior ECGs had not suggested ischaemia. The subsequent unremarkable coronary angiogram added weight to the likely thromboembolic nature of the infarction, potentially via multiple microemboli being thrown off the LV thrombus.

The precise timing of the presumed embolisation to our patient’s coronary circulation is unclear, and the absence of overt ischaemic cardiac symptoms suggests this may have been a relatively prolonged, subacute process. Earlier recognition of the thrombotic nature of this event may have prevented myocardial injury if embolic showers continued into her inpatient stay.

**Case report - Key learning points:**

1. In addition to presentation with common thromboses such as DVT, stroke or PE, patients with antiphospholipid syndrome may also develop thrombosis at unusual sites such as the hepatic circulation, mesenteric vessels or within the heart, or alternatively with widespread thrombi in multiple organ systems as part of a catastrophic APLS presentation.
2. In patients with a history of antiphospholipid syndrome and/or associated rheumatic diseases such as SLE presenting with acute symptoms, specific and careful consideration should be given to arterial and venous thrombotic events, at common and rarer sites.
3. A lower threshold for imaging these suspected sites compared to patients without known thrombophilia may be necessary.
4. Lupus patients’ full antiphospholipid antibody status should always be checked at diagnosis to inform decision-making at any subsequent acute presentation, or if not previously performed, as soon as possible when presenting with acute symptoms potentially attributable to thromboembolic disease.
5. Ischaemic microthrombotic events may occur subacutely in antiphospholipid syndrome without symptoms until evidence of organ failure develops.

